# Magnetic Fluorescence Molecularly Imprinted Polymer Based on FeO_x_/ZnS Nanocomposites for Highly Selective Sensing of Bisphenol A

**DOI:** 10.3390/polym11071210

**Published:** 2019-07-19

**Authors:** Xin Zhang, Shu Yang, Weijie Chen, Yansong Li, Yuping Wei, Aiqin Luo

**Affiliations:** 1 School of Life Science and Technology, Nanyang Normal University, Nanyang 473061, China; 2 School of Life Science, Beijing Institute of Technology, No.5 Zhongguancun South Street, Beijing 100081, China

**Keywords:** molecularly imprinted polymer, fluorescence magnetic polymer, fluorescence sensing, magnetic FeO_x_/ZnS quantum dots, bisphenol A

## Abstract

In this study, magnetic fluorescence molecularly imprinted polymers were fabricated and used for the selective separation and fluorescence sensing of trace bisphenol A (BPA) in environmental water samples. The carboxyl-functionalized FeO_x_ magnetic nanoparticles were conjugated with mercaptoethylamine-capped Mn^2+^ doped ZnS quantum dots to prepare magnetic FeO_x_ and ZnS quantum dot nanoparticles (FeO_x_/ZnS NPs). Additionally, molecular imprinting on the FeO_x_/ZnS NPs was employed to synthesize core-shell molecularly imprinted polymers. The resulting nanoparticles were well characterized using transmission electron microscopy, Fourier transform infrared spectra, vibrating sample magnetometer and fluorescence spectra, and the adsorption behavior was investigated. Binding experiments showed that the molecularly imprinted FeO_X_/ZnS NPs (FeO_x_/ZnS@MIPs) exhibited rapid fluorescent and magnetic responses, and high selectivity and sensitivity for the detection of bisphenol A (BPA). The maximum adsorption capacity of FeO_x_/ZnS@MIPs was 50.92 mg·g^−1^ with an imprinting factor of 11.19. Under optimal conditions, the constructed fluorescence magnetic molecularly imprinted polymers presented good linearity from 0 to 80 ng mL^−1^ with a detection limit of 0.3626 ng mL^−1^ for BPA. Moreover, the proposed fluorescence magnetic polymers were successfully applied to on-site magnetic separation and real-time fluorescence analysis of target molecule in real samples.

## 1. Introduction

Multifunctional polymers with special physical and chemical properties (such as optical, electrical, thermal, chirality, and magnetic characteristics) have drawn increasing attention due to their potential application in numerous areas [1,2,3,4]. As a kind of luminescent nanomaterial, quantum dots (QDs) have high quantum yields, excellent photostability, broad excitation and narrow symmetric emission spectrum, and a size-dependent band gap, therefore, they have attracted considerable attention [5,6]. On the other hand, magnetic nanoparticles (MNPs), which are used as an important magnetic nanomaterial, have great potential applications in magnetic resonance imaging (MRI), drug delivery, catalysis, chemo/biosensors, and medicine diagnosis [7,8,9,10]. MNPs have a large surface area and high mass transference based on their size, which enables them to promote fast electron transfer [11,12,13]. Moreover, MNPs can be directly separated; this facilitates ultra-trace analyte enrichment without going through centrifugation and filtration steps in bioassay, and improves the detection efficiency and sensitivity.

By combining quantum dots with magnetic nanocrystals, an advanced nanocomposite polymer with excellent functionalities can be prepared, which simultaneously integrates the optical and magnetic properties [14,15]. A magnetic nanoparticle coupled with quantum dots can be directly separated and is then able to generate readable optical signals for analysis. The ability to combine the purification process with the detection procedure in one step means that magnetic quantum dot polymers have a promising future and novel applications in bio-detection, biomedicine, drug delivery, and environmental monitoring [16,17,18]. However, when dealing with a complex matrix, the synthesized magnetic quantum dots demonstrate nonspecific binding in the separation process, which results in high level background fluorescence response and restricts their specificity and sensitivity for analysis. To enhance the specificity and sensitivity of magnetic quantum dots, a molecular imprinted polymer (MIP) layer can be loaded on the surface of the magnetic quantum dot nanocomposite to tailor the selectivity of analytes using molecular imprinting technology [19,20,21,22].

Molecular imprinting is a well-established technique to design an artificial molecular recognition unit that involves polymerizing functional monomers in the presence of template molecules [23]. MIPs exhibit excellent selectivity and affinity with the template molecules [24]. The MIP layer coated on the surface of magnetic quantum dots will introduce the selectivity recognition sites to the nanocomposites and prevent interfering molecules from binding with the nanocomposites. MIPs have been introduced as promising recognition elements with high selectivity for detecting trace analyte. Zhang et al. developed imprinted polymer coating CdTe quantum dots for specific recognition of BHb [25]. Zhao et al. prepared ZnS QDs-based molecularly imprinted polymer composite nanospheres for fluorescent quantification of pesticides [26]. Zhao et al. used molecularly imprinted water-soluble CdTe QDs for Listeria monocytogenes detection in food samples [27]. As CdTe QDs suffer from bio-compatibility issues, ZnS QDs based MIPs with low biotoxicity are preferable [28]. Furthermore, magnetic nanoparticles involving fluorescent multifunctional nanoparticles can facilitate magnetic separation, also the fluorescent response is quicker because the magnetic nanoparticles promote faster electron transfer. So together with MIPs’ selectivity, this novel multifunctional sensor is expected to demonstrate improved properties for trace target detection.

In this work, an innovative magnetic fluorescent sensor based on FeO_x_/ZnS@MIPs was prepared for the separation and detection of trace BPA in complex samples. Bisphenol A (2,2-bis(4-hydroxyphenyl)propane) is an estrogenic endocrine disruptor. It is a common chemical that has been extensively used in the manufacture of polycarbonate plastics and epoxy resins for the linings of food, beverage packaging, and other consumer products [29]. Increasing evidence indicates that BPA can migrate from containers into foods and beverages, and trace residue levels of BPA can be released into environmental water through diffusion or degradation, which carries a high risk of causing adverse effects on human reproductive health and the ecosystem [30,31]. Because of BPA’s ubiquity in nature and its potential implications in human health and the ecological environment, various analytical methods have been developed for monitoring or detecting BPA [32,33,34,35,36,37,38,39,40,41]. However, many of those analytical techniques for BPA need sophisticated instrumentation, and the procedures involve pre-concentration, extraction, purification or derivatization [42,43,44]. Those methods are costly and time-consuming, and require well trained and experienced personnel to guarantee the accuracy of results. Therefore, it would be highly advantageous to develop a low-cost, selective method for the convenient and rapid separation and detection of BPA. The prepared FeO_x_/ZnS@MIP enables fluorescence analysis and the efficient separation of BPA without any expensive instruments or time-consuming procedures. Based on the fluorescence quenching being proportional to the concentration of the target molecule, FeO_x_/ZnS@MIP was successfully applied to the direct fluorescence sensing of BPA without any further pretreatment. Notably, FeO_x_/ZnS@MIP has the advantage that it can be easily recycled and rapidly removed from the trace contaminant in the environment. The FeO_x_/ZnS@MIPs could specifically bind and magnetically enrich trace BPA, which avoids interfering substances in complex matrices and enhances the detection efficiency and sensitivity. Additionally, the proposed methods for the preparation of FeO_x_/ZnS@MIPs exhibits promising potential to isolate and detect proteins and other target molecules in biology detection applications.

## 2. Materials and Methods 

### 2.1. Materials

All of the solvents and chemicals used were of analytical grade and were used without further purification. Zinc sulfate (ZnSO_4_·7H_2_O), sodium sulphide (Na_2_S·9H_2_O), manganese chloride (MnCl_2_·4H_2_O), ferric chloride crystal (FeCl_3_·6H_2_O), anhydrous sodium acetate (NaAc) and trisodium citrate dihydrate (Na_3_Cit·2H_2_O) were purchased from Beijing Chemical Works (Beijing, China). Bisphenol A, 4,4′-bisphenol (BP) and ethylene glycol (EG) were obtained from Sinopharm Chemical Reagent Co. Ltd. (Beijing, China). 1.1-Bis (4-hydroxyphenyl) cyclohexane (BPZ) was received from Tokyo Chemical Industry Co. Ltd. (Tokyo, Japan). Mercaptoethylamine (MEA), 1-ethyl-3-(3-dimethylaminopropyl) carbodiimide (EDC), *N*-hydroxysuccinimide (NHS) were purchased from Macklin Biochemical Co. Ltd. (Shanghai, China). Tetraethyl silicate (TEOS), 4-tert-butylphenol (PTBP), 3-aminopropyltriethoxylsilane (APTES) were purchased from Beijing J&K Chemical Technology Co. Ltd. (Beijing, China). All solutions were prepared with double deionized water (DDW).

### 2.2. Instrumentation

All the fluorescence measurements were recorded using a spectrofluorometer (Shimadzu, RF-6000, Kyoto, Japan). BPA adsorption data were recorded on a UV–Vis spectrophotometer (Shimadzu, Kyoto, Japan). The magnetization measurements of the FeO_x_ MNPs, MNP/QDs and MNP/QD@MIPs were carried out using a vibrating sample magnetometer (VSM, Lake Shore 7307, Columbus, OH, USA) at room temperature. The morphology of the QDs was characterized by a JEM-2100F high resolution transmission electron microscopy (TEM, JEOL, Tokyo, Japan). The shape and structures of the magnetic nanomaterials were examined using a H-800 transmission electronic microscopy (Hitachi, Tokyo, Japan). The Fourier transform infrared (FT-IR) spectra were determined on a Vertex 70v spectrometer (Bruker, Karlsruhe, Germany).

### 2.3. Synthesis of Amino-Modified ZnS: Mn^2+^ QDs and Carboxyl-Functionalized MNPs 

The MEA-modified Mn^2+^-doped ZnS QDs (ZnS: Mn^2+^ QDs@MEA) and the carboxyl-functionalized FeO_x_ magnetic nanoparticles (FeO_x_@COOH MNPs) were synthesized based on previously reported methods with some modification [45,46,47]. The preparation procedures are presented in the Supplementary Materials.

### 2.4. Synthesis of FeO_x_/ZnS Nanoparticles

The FeO_X_/ZnS NPs was synthesized via an EDC/NHS reaction process. Briefly, 20 mg of FeO_x_@COOH MNPs were first dispersed in 20 mL citrate buffer solution (0.02 mol L^−1^, pH = 6.4) to prepare magnetic fluids. Then, 2 mL of magnetic fluids were added in 50 mL EDC/NHS activating agent (1:1.2 g L^−1^). The above mixture was stirred for 2 h, followed by 30 mg of as-prepared amino-modified QDs being added and incubated at 30 °C for 20 h. The brown solutions were collected by magnetic decantation, and washed with water and ethanol to remove the residual substance, then redispersed in buffer solution for further use.

### 2.5. Fabrication of FeO_x_/ZnS@MIPs

The FeO_x_/ZnS@MIPs for BPA were prepared through molecular imprinting on the surface of magnetic fluorescence nanoparticle. For the fabrication of FeO_x_/ZnS@MIPs, 10 mL of a methanol solution containing 10 mg BPA and 60 μL APTES were first added to a 25 mL flask and stirred for 30 min. Then, 60 mg of the as-prepared FeO_x_/ZnS nanoparticle and 100 μL TEOS were added in sequence. After continuously stirring for another 30 min, 2.5 mL of 5% NH_3_·H_2_O (the catalyst) was added. The solution was deoxygenated by purging with nitrogen and stirred overnight. Non-imprinted polymers (FeO_x_/ZnS@NIPs) were prepared by the same procedure but without the addition of BPA. The resultant products were magnetically decanted and washed with a mixture of methanol and acetic acid (9:1, *v*/*v*) to remove the template molecules until the fluorescence intensity of FeO_x_/ZnS@MIPs was not changed and was similar to that of the FeO_x_/ZnS@NIPs ones.

### 2.6. Fluorescence Sensing of BPA 

All of the fluorescence measurements were examined using the same condition. The excitation wavelength was set to be 311 nm with the fluorescence intensity recorded at 586 nm. The recording fluorescence emission spectrum was ranged from 350 to 700 nm.

The standard solution of bisphenol A was prepared first. Then, various samples in concentrations ranging from 1.0 to 80 ng mL^−1^ were made by diluting with citrate buffer solution (0.02 mol L^−1^, pH = 6.4). Two milligrams of FeO_x_/ZnS@MIPs or FeO_x_/ZnS@NIPs were dispersed in 10 mL testing samples. After incubating the samples for 5 min at room temperature, the changes in the fluorescence intensity of the solutions were recorded using a spectrofluorometer. This fluorescence quenching model was in accordance with the Stern–Volmer equation [48]:

(1) $$F_{0}/F=1+K_{\mathrm{SV}}C_{\mathrm{BPA}}$$

where *F*_0_ is the initial fluorescence intensity in the absence of the BPA, *F* is the fluorescence intensity in the presence of the BPA, *K*_SV_ is the quenching constant, and *C*_BPA_ is the concentration of BPA.

### 2.7. Binding Selectivity

The selectivity experiments were carried out with PTBP, BP and BPZ as structural analogs of BPA to run a batch rebinding test. Briefly, PTBP, BP and BPZ samples in the concentration ranged from 1.0 to 80 ng mL^−1^ and were made by diluting with citrate buffer solution. Two milligrams of FeO_x_/ZnS@MIPs or FeO_x_/ZnS@NIPs were dispersed in 10 mL testing samples and incubated for 5 min. Then the changes in the fluorescence intensity of different samples were recorded, and the imprinting factor (IF) and selectivity coefficient (SC) were used to evaluate the selectivity properties of FeO_x_/ZnS@MIPs and FeO_x_/ZnS@NIPs toward the template BPA and structural analogs [25]:

(2) IF=KMIPKNIP

(3) SC=IFIF′

where *K*_MIP_ and *K*_NIP_ are the slopes of the linear equation of FeO_x_/ZnS@MIPs and FeO_x_/ZnS@NIPs with the target molecule, respectively, IF and IF’ are the imprinting factor for template BPA and structural analogs, respectively.

### 2.8. Analysis of Real Samples

The prepared FeO_x_/ZnS@MIP was directly applied to the detection of BPA in drinking water, tap water, and lake water. The drinking water was commercial pure water purchased from the market. The tap water was collected from the laboratory. The lake water samples were collected from three different lakes (located in Beijing, China). All the samples were filtered through a 0.45 μm filter and stored at 4 °C.

The detection strategy was divided into two steps: (a) the enrichment and separation of BPA and (b) fluorescence detection (Figure S1). Firstly, the FeO_x_/ZnS@MIPs were dispersed in the water samples. The target BPA molecule was specifically bound onto the MIP layer of the FeO_x_/ZnS@MIPs after incubating at room temperature. Secondly, the fluorescence quench of FeO_x_/ZnS@MIPs in each sample was recorded and the concentration of analytes in the samples was calculated. There were no other pretreatment procedures employed in the sample preparation. To evaluate the developed method, a recovery test was carried out by using the samples spiked with BPA standard solution.

## 3. Results and Discussion 

### 3.1. Synthesis of the FeO_x_/ZnS@MIPs 

**Figure 1:**
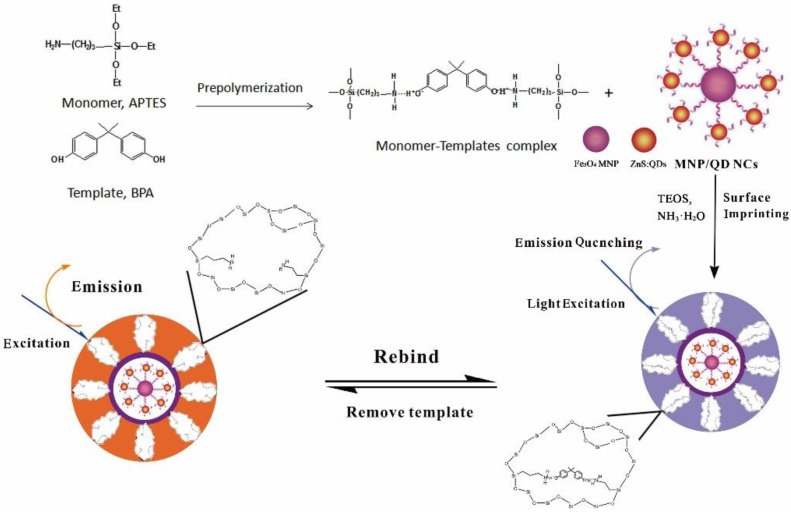
Schematic illustration of the process for the fabrication of the FeO_x_/ZnS@MIP-based sensor. BPA: bisphenol A.

The design of the FeO_x_/ZnS@MIPs was mainly based on coating the molecularly imprinted polymer layer on the surface of FeO_x_/ZnS NPs. Figure 1 illustrates the two major synthetic steps of the proposed fluorescence sensing polymer. In the first step, mercaptoethylamine was grafted onto the surface of the Mn^2+^-doped ZnS QDs. The mercapto of MEA was tightly bound at the surface of the bare QDs through ligand competition. The introduction of amino group to the QDs not only increases the water dispersion ability of QDs, but also provides the possibility of being combined with FeO_x_ magnetic nanocrystals. FeO_x_ magnetic nanocrystals were successfully synthesized by a modified solvothermal method. The –COOH was grafted onto the surface of FeO_x_ in just one synthesis step. The carboxyl-functionalized FeO_x_ NPs were conjugated with MEA capped QDs to prepare the multifunctional nanocomposites. The FeO_x_/ZnS NPs integrated the distinct properties of the optical characteristics of QDs, and the magnetic separation ability of MNPs through an EDC/NHS reaction process. In the second step, APTES was chosen as a functional monomer that had noncovalent interactions with bisphenol A [49]. The monomer (APTES) interacted with the template molecule (BPA) through a hydrogen bond to form a “pre-polymerization” complex (Figure 1). The resultant FeO_x_/ZnS NPs were used as substrate, TEOS and NH_3_·H_2_O were used as the crosslinker and catalyst, respectively. The pre-polymerization complex was subsequent immobilized on the surface of FeO_x_/ZnS NPs through a facile molecular imprinting process. The imprinting layer was coated on the FeO_x_/ZnS NPs to produce a “core-shell” structure. This core-shell composite provides selectivity to the template and prevents other interfering molecules from contacting the FeO_x_/ZnS NPs. After the removal of the template molecule BPA, the MIP layer with imprinted cavities complementary to the BPA in size, shape, and functional groups was obtained. The quantum yield (QY) of the FeO_x_/ZnS NPs was 20.6%, as calculated by equation S1. The resultant FeO_x_/ZnS@MIPs, as an ideal candidate material, was able to be used as a multifunctional sensor for high selectivity and sensitivity magnetic separation and the fluorescent detection of target BPA. 

### 3.2. Characterization

**Figure 2:**
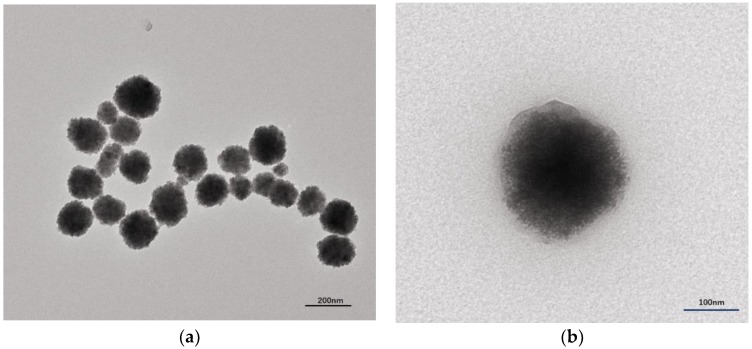
TEM images of (**a**) FeO_x_/ZnS NPs and (**b**) FeO_x_/ZnS@MIPs.

The morphology of the obtained FeO_x_/ZnS NPs and FeO_x_/ZnS@MIPs was investigated by transmission electron microscopy. As shown in Figure 2a, the particles of FeO_x_/ZnS NPs were spherical in morphology, and they were about 135.2 ± 16.7 nm. The magnetic nanoparticles were tightly surrounded by ZnS: Mn^2+^ QDs@MEA through the EDC/NHS reaction, which created a rough surface on the FeO_x_/ZnS NPs. After the molecular imprinting process, the size of the resulting FeO_x_/ZnS@MIPs increased to about 198.6 ± 13.5 nm and displayed a smooth surface (Figure 2b). As can be seen, an MIP layer had been well coated on the surface of FeO_x_/ZnS NPs, and an interface could be clearly distinguished between the inner FeO_x_/ZnS NPs core and the imprinting polymer shell.

**Figure 3:**
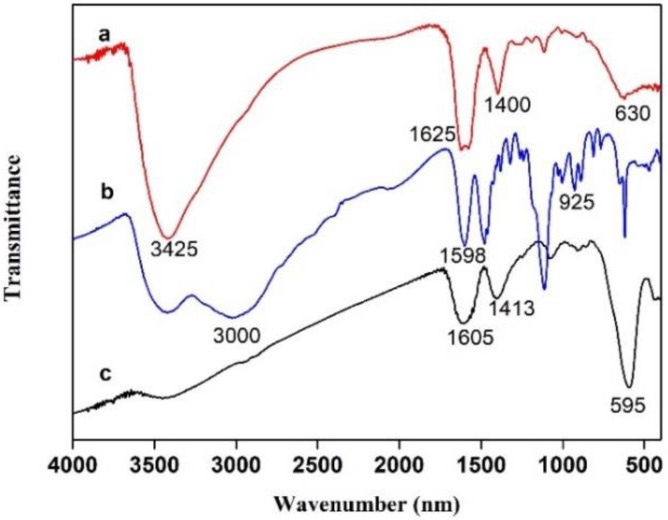
FT-IR spectra of (**a**) FeO_x_/ZnS NPs, (**b**) ZnS: Mn^2+^ QDs@MEA and (**c**) FeO_x_@COOH nanoparticles.

The structure of ZnS: Mn^2+^ QDs@MEA, FeO_x_@COOH and FeO_x_/ZnS NPs was analyzed by FT-IR spectroscopy. As shown in Figure 3, the characteristic Fe–O bands were present in FeO_x_/ZnS (632 cm^−1^) and FeO_x_@COOH MNPs (594 cm^−1^), respectively. Peaks at 1598 cm^−1^ (amino groups), 925 cm^−1^ (bending vibration) and the disappeared peaks at 2550–2670 cm^−1^ (S–H thiol group) in the curve b indicated that the MEA was modified on the surface of ZnS: Mn^2+^ QDs through covalent bonds formed between thiols and Zn^2+^ surface atoms [50]. The Peak at 1413 cm^−1^ corresponded to C–O on the FeO_x_, and a peak at 1605 cm^−1^ corresponded to the vibration of water molecules adsorbed on Fe_3_O_4_ [13]. Compared with the ZnS: Mn^2+^ QDs@MEA and FeO_x_@COOH (curve b and c), FeO_x_/ZnS NPs (curve a) showed characteristic peaks at 3425 cm^−1^ (N–H stretching vibration), 1625 cm^−1^(N–H bend), and 1400 cm^−1^(C–N stretching vibration), revealing that an amide bond was formed. The results suggested that the ZnS: Mn^2+^ QDs@MEA and FeO_x_@COOH were successfully combined through an EDC/NHS reaction process.

**Figure 4:**
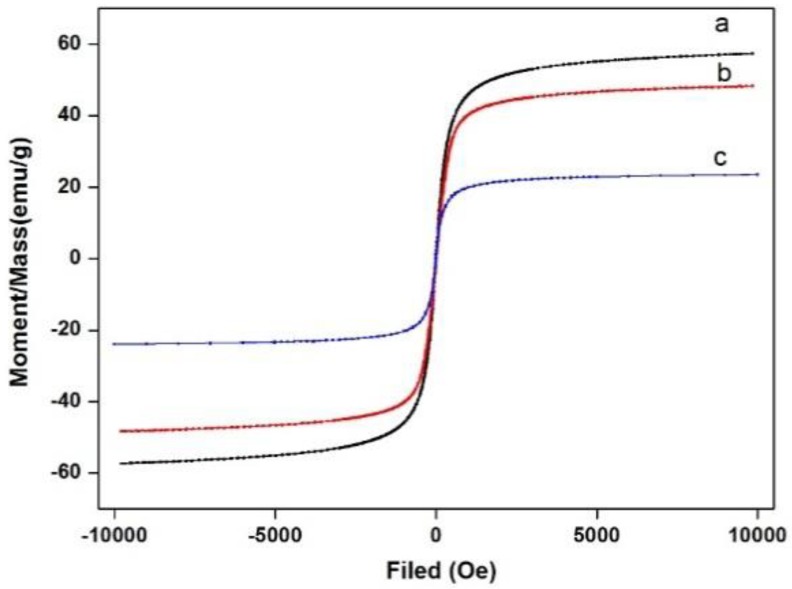
The magnetic hysteresis loops of (**a**) FeO_x_@COOH magnetic nanoparticles, (**b**) FeO_x_/ZnS NPs, and (**c**) FeO_x_/ZnS@MIPs.

To study the influence of surface modification on the magnetic behavior of FeO_x_@COOH MNPs, FeO_x_/ZnS NPs and FeO_x_/ZnS@MIPs, the VSM magnetization curves of the as-prepared magnetic materials were compared in Figure 4. As shown in Figure 4, all of the magnetic hysteresis loops of the magnetic materials displayed a typical super-paramagnetic characteristic and high magnetization, and the magnetization saturation values of FeO_x_@COOH MNPs, FeO_x_/ZnS NPs, and FeO_x_/ZnS@MIPs were about 57.348, 45.1033, 24.5796 emu g^−1^, respectively. The magnetization saturation value of prepared NPs was lower than that of pure Fe_3_O_4_ magnetite (about 80 emu g^−1^), which is due to some mixed Fe_2_O_3_ that lowers the magnetization saturation. The as-prepared magnetic NPs still demonstrated a strong magnetic response and FeO_x_ was used to represent the mixture to describe the magnetic behavior in this paper. The magnetization saturation values of FeO_x_/ZnS@MIPs were lower than that of FeO_X_@COOH MNPs, which may be attributed to the MIP shell on the surfaces of FeO_X_/ZnS NPs. Additionally, the as-prepared magnetic nanocomposites also showed a rapid magnetic response to the applied magnetic field. Once the magnetic field was removed, the magnetic nanocomposites homogeneously and quickly redispersed with a slight shake. This demonstrates that the FeO_X_/ZnS@MIPs possess rapid magnetic responsivity and good dispersibility, which enables them to be practically used for the rapid separation or enrichment of analyte in complex samples.

The optical properties of ZnS: Mn^2+^ QDs@MEA and FeO_x_/ZnS NPs were investigated by UV–Vis spectra and fluorescence spectra. As shown in Figure S2a,b, the QDs and FeO_x_/ZnS NPs exhibit semblable absorption spectrum, indicating the QDs successfully combined with the FeO_x_ MNPs. Compared with ZnS: Mn^2+^ QDs@MEA, the absorption peak of FeO_x_/ZnS NPs is less pronounced, which attributed to the broad and strong absorption of the combined Fe_3_O_4_ MNPs [51]. The ZnS: Mn^2+^ QDs@MEA and FeO_x_/ZnS NPs show well-resolved emission spectra, with both of the maximum emission peaks located at 586 nm. The characteristic emission peak of FeO_x_/ZnS NPs corresponded to the ZnS: Mn^2+^ QDs@MEA, implying that the fluorescence property was not significantly affected in the EDC/NHS reaction process. Both samples possess a narrow and symmetrical emission peak and large Stokes shift, which is more suitable for fluorescence labeling in vivo or biosensing target analytes in complex biological samples.

### 3.3. Fluorescence Response to Time and Adsorption Kinetics 

The effect of reaction time on the fluorescence intensity and the adsorption kinetics curves of BPA onto FeO_x_/ZnS@MIPs are presented in Figures S3 and S4, respectively. As shown in Figure S3, the fluorescence intensity of FeO_x_/ZnS@MIPs showed a rapid decrease in the first 2 min and achieved stable fluorescence intensity after being incubated for 5 min, which corresponded closely to the adsorption equilibrium time (Figure S4). The adsorption equilibrium for the FeO_x_/ZnS@NIPs emerged at 4 min in the experiment; however, the fluorescence quenching and adsorption amount of FeO_x_/ZnS@NIPs was lower than that of the MIP ones. This is due to the non-specific binding cavities that are formed in the FeO_x_/ZnS@NIPs’ synthesis process, the non-specific adsorption was dominant in the FeO_x_/ZnS@NIPs, which resulted in a lower binding capacity and lower fluorescence quenching. These results verified that the optimal reaction time of the FeO_x_/ZnS@MIPs for detecting BPA was 5 min.

### 3.4. Binding Performance 

**Figure 5:**
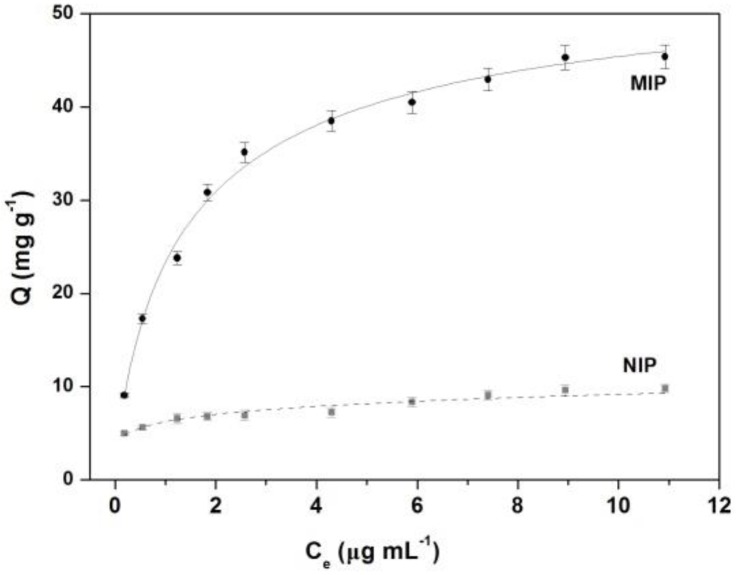
The adsorption isotherm and Langmuir fit of FeO_x_/ZnS@MIPs. Experimental conditions: citrate buffer solution (0.02 mol L^−1^, pH = 6.4), room temperature.

The adsorption isotherm was investigated through a batch affinity adsorption experiment of FeO_x_/ZnS@MIPs. A series of BPA standard samples were used to evaluate the adsorption isotherms of BPA on the FeO_x_/ZnS@MIPs and FeO_x_/ZnS@NIPs. As shown in Figure 5, the adsorption capacity of FeO_x_/ZnS@MIPs increased quickly with the increasing concentration of BPA. The FeO_x_/ZnS@MIPs exhibited a higher binding amount of BPA than that of the FeO_x_/ZnS@NIPs. Because of the 3D-imprinted cavities in FeO_x_/ZnS@MIPs, which possessed better chemical and structure matching with the template BPA, a large number of empty recognition cavities were available in the FeO_x_/ZnS@MIPs, which enabled BPA molecules to easily enter, and resulted in excellent adsorption capacity. To further study the adsorption performance of FeO_x_/ZnS@MIPs, the experimental data were fitted with Langmuir and Freundlich isotherm models. Fitting results showed that the binding properties were best described by the Langmuir isotherm model (R = 0.9930), which revealed that the adsorption behavior of FeO_x_/ZnS@MIPs was basically monolayer adsorption onto a surface with a homogeneous system. The Langmuir isotherm equation of FeO_x_/ZnS@MIPs for BPA was *C*_e_/*Q* = 0.2302 + 0.01964 *C*_e_, and the maximum adsorption capacity calculated by the Langmuir isotherm model was 50.92 mg g^−1^.

### 3.5. Fluorescence Sensing of BPA

**Figure 6:**
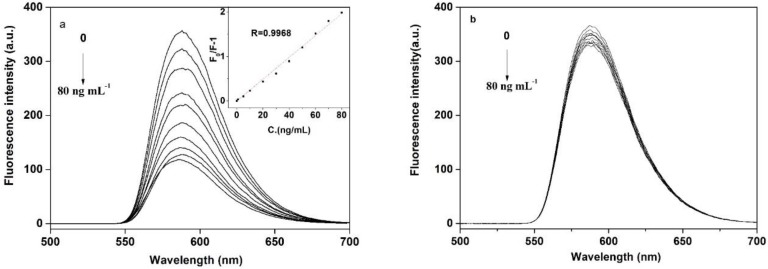
Fluorescence emission spectra of (**a**) FeO_x_/ZnS@MIPs and (**b**) FeO_x_/ZnS@NIPs with increasing concentrations of BPA. Inset graphs: the linear calibration of the fluorescence intensity change (*F*_0_/*F*) versus BPA concentration. Experimental conditions: BPA (0, 5, 10, 20, 30, 40, 50, 60, 70, 80 ng mL^−1^), citrate buffer solution (0.02 mol L^−1^, pH = 6.4), room temperature.

To maintain the fluorescence stability of the FeO_x_/ZnS@MIPs, all experiments were performed in citrate buffer solution at pH 6.4. The typical fluorescence quenching of imprinted FeO_x_/ZnS NPs in the concentration of BPA ranged from 1.0 to 80 ng mL^−1^, as shown in Figure 6. It can be seen that the FeO_x_/ZnS@MIPs showed noticeable fluorescence emission responses to different concentrations of BPA. As a control, the FeO_x_/ZnS@NIP was slightly quenched by BPA because of there being no specific recognized site on the surface of the NIPs; thus, fewer BPA molecules were bound by non-specific interactions. In contrast, the fluorescence emission of the FeO_x_/ZnS@MIPs quenched gradually with the increasing concentration of BPA. The fluorescence intensity of FeO_x_/ZnS@MIPs in the presence of BPA (80 ng mL^−1^) was only 32.7% compared to that in the absence of BPA. The experiment results showed that the fluorescence quenching of the FeO_x_/ZnS@MIPs depended on the specific binding with template BPA. Therefore, FeO_x_/ZnS@MIPs can be used for the detection of trace BPA. Under optimal conditions, the plot of fluorescence intensity change (*F*_0_/*F*-1) versus the concentration of BPA (ng mL^−1^) showed a good linear relationship. The linear relationship between fluorescence intensity and the BPA concentrations was in the range of 0 to 80 ng mL^−1^ with a correlation coefficient of 0.9968 (n = 11). The corresponding limit of detection (LOD) following the IUPAC criteria (3σ/*S*) was calculated as 0.3626 ng mL^−1^. This value of LOD was lower than the permitted maximum residue limits of BPA in drinking water (10 ng mL^−1^, GB 5749-2006) [40], indicating that the as-prepared FeO_x_/ZnS@MIPs can be employed for environmental and drinking water safety monitoring. The comparison of this performance with other reported analytical methods for BPA detection is shown in Table 1. The results showed that the strategy presented is more rapid, selective and sensitive than the traditional methods. The proposed magnetic/fluorescence molecularly imprinted polymer coated FeO_x_/ZnS nanocomposites not only have the merits of convenience and low cost, but also have high selectivity and a comparable or lower limit of detection, which makes them a promising fluorescent sensor for the selective and sensitive detection of target molecules.

### 3.6. Rebinding Selectivity

The specific recognition ability of FeO_x_/ZnS@MIPs was further investigated. The imprinting factor (IF) and selectivity coefficient (SC) were used to evaluate the selectivity of the FeO_x_/ZnS@MIPs towards the template BPA and structural analogs (Figure S5). The imprinting factor is the ratio of the *K*_MIP_ and *K*_NIP_ (*K* is the slope of the linear equation), and the selectivity coefficient is the ratio of IF for the template molecule and structural analogs (Table S1).

**Figure 7:**
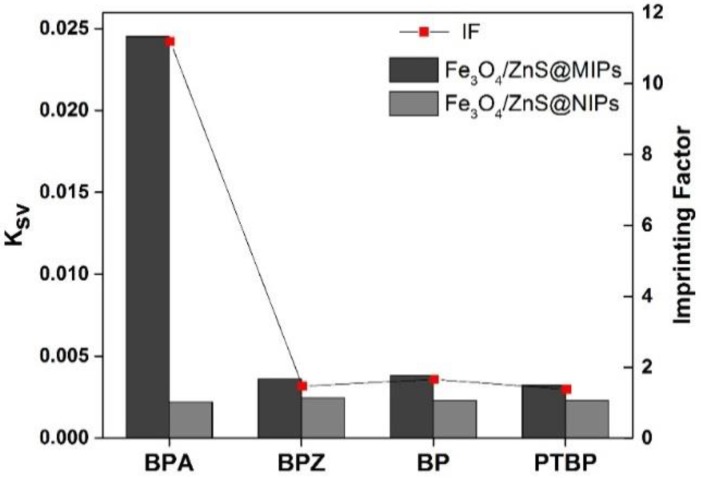
The quenching constant (*K*sv) and the imprinting factor (IF) of BPA, 1.1-Bis (4-hydroxyphenyl) cyclohexane (BPZ), 4,4′-bisphenol (BP) and 4-tert-butylphenol (PTBP).

As shown in Figure 7, the BPA molecule exhibited a significant fluorescence quenching effect on the FeO_x_/ZnS@MIPs. Table S1 shows that the imprinting factor for BPA was 11.19, which is much larger than that of BPZ, BP, and PTBP (1.47, 1.66, and 1.39, respectively). This was because the template BPA had more access to the recognition cavities of FeO_x_/ZnS@MIPs, which were formed in the imprinting process. Conversely, there are no complementary binding sites formed between FeO_x_/ZnS@NIPs and analyte molecules. The BPA and its analogs bound on the FeO_x_/ZnS@NIPs are mainly bound through non-specific interactions. Therefore, the *K*_NIP_ for the BPA and structural analogs (BPZ, BP and PTBP) was almost the same, and a lower fluorescence quenching effect on the FeO_x_/ZnS@NIPs was observed (Figure 7). The experimental results showed that the selectivity of the MIPs for template BPA was much higher than that of structural analogs, indicating that a molecular imprinting process can greatly enhance the selectivity of FeO_x_/ZnS@NPs.

### 3.7. Analysis of Real Samples

In order to demonstrate the practical applicability of FeO_x_/ZnS@MIPs in real samples, different water samples (drinking water, tap water, and lake water) were used to evaluate the separation effectiveness and detection accuracy. The detection strategy is shown in Figure S1.

As shown in Table 2, the detected BPA values in the lake sample were 1.52, 7.39 and 6.54 ng mL^−1^, respectively. BPA was not found in drinking water and tap water at the detection limit level of 0.36 ng mL^−1^. The recovery rates of the present FeO_x_/ZnS@MIPs ranged from 90.8% to 103.1%, and the relative standard deviation (RSD) values were between 2.7% and 5.4% (n = 3). The above results proved that the prepared FeO_x_/ZnS@MIPs could be successfully applied to the magnetic separation and fluorescence detection of the target molecule in practical applications.

### 3.8. Recyclability and Stability 

The recyclability test was done by observing the changes in fluorescence intensity with six rebind/elution cycles. As shown in Figure S6a, the fluorescence properties of the FeO_x_/ZnS@MIPs performance slightly decreased in five regeneration cycles. However, the fluorescence intensity decreased 25.5% and 31.1% in the next two regeneration cycles, respectively. This indicates that too much binding/removing template will affect the structure of the FeO_x_/ZnS@MIPs. The stability of FeO_x_/ZnS@MIPs was evaluated by measuring the initial fluorescence intensities and those after 1–35 days of storage under dark conditions (Figure S6b). It can be seen that the fluorescence intensity showed no significant change after being stored for a long time. These results suggest that FeO_x_/ZnS@MIPs have good regeneration capacity and stability.

## 4. Conclusions

In this study, a novel approach for the preparation FeO_x_/ZnS@MIPs has been successfully developed. The fabricated core-shell nanocomposites integrated selective magnetic separation and fluorescence analysis, therefore, they can be used for direct magnetic separation and the selective detection of trace BPA in complex environmental or biological matrices. A series of rebinding experiments showed that the proposed FeO_x_/ZnS@MIP-based sensor has better selectivity and sensitivity for target BPA than its analogues. The FeO_x_/ZnS@MIPs also showed good reusability and stability in practical applications. Moreover, by changing the template molecule, this novel approach to multifunctional sensor preparation can be expanded to other organic molecules or protein biomolecules. Such superior optical and physical merits endow the FeO_x_/ZnS@MIPs with promising potential in food, environmental, clinical diagnostic, and biomedical research.

## Figures and Tables

**Table 1:** The comparison of the results of different analytical techniques for the detection of BPA.

Detection Technique	Liner Range (ng mL^−1^)	LOD (ng mL^−1^)	Imprinting Factor (IF)	References
MIP/SERS	5.0 × 10^2^–2.28 × 10^4^	120	1.90	[32]
MIP-coated Au NCs	0–2.98 × 10^3^	22.8	2.25	[33]
Colorimetric method	35–1.40 × 10^2^	0.11	/	[34]
Fluorescent aptasensor	10–80	1.86	/	[35]
MIP-coated CDs	22.8–9.60 × 10^2^	6.84	/	[36]
Voltammetric sensor	11.4–2.28 × 10^3^	1.82	/	[37]
Imprinting SiO_2_-coated CdTe NPs	11.4–2.28 × 10^2^	1.368	5.2	[38]
Ferrocenyl-based MIP	1.07–1.8	0.7296	1.84	[39]
Colorimetric sensor-MIP	2.28–2.28 × 10^2^	1.41	/	[40]
Electrochemical sensor-MIP	18.4–2.28 × 10^4^	8.66	/	[41]
MIP-SPE/HPLC	0.11–22.8	0.11	1.97	[42]
GC-MS	1–200	1.0	/	[43]
FeO_x_/ZnS@MIPs	0–80	0.3626	11.19	This Work

SERS: surface enhanced Raman scattering; GC-MS: gas chromatography-mass spectrometer; SPE: solid-phase extraction; Au NCs: gold nanoclusters; CDs: carbon dots; LOD: limit of detection.

**Table 2:** Detection of BPA in real water samples using FeO_x_/ZnS@MIPs sensor.

Sample	Detected (ng mL^−1^)	Added (ng mL^−1^)	Measured (ng mL^−1^) ^a^	Recovery (%)	RSD (n = 3, %)
Drinking water	n.d ^a^	2.28	2.20	96.5	3.5
		22.80	22.44	98.4	2.7
Tap water	n.d	2.28	2.12	93.0	4.1
		22.80	21.15	92.8	3.3
Lake water sample1	1.52	2.28	2.07	90.8	4.2
		22.80	20.73	90.9	4.9
Lake water Sample2	7.39	2.28	2.35	103.1	5.3
		22.80	22.25	97.6	4.5
Lake water Sample3	6.54	2.28	2.31	101.3	5.4
		22.80	21.98	96.4	3.8

^a^ Average value; RSD: relative standard deviation.

## Supplementary Materials

The supplementary materials are available online at https://www.mdpi.com/2073-4360/11/7/1210/s1.
