# Supplementary material for: Magnetic Fluorescence Molecularly Imprinted Polymer Based on FeOx/ZnS Nanocomposites for Highly Selective Sensing of Bisphenol A
Source: Polymers (Basel). 2019 Jul 19;11(7):1210. doi: 10.3390/polym11071210 (PMC6680805; doi:10.3390/polym11071210)
Supplement: Supplementary file 1 [file polymers-11-01210-s001.pdf]

Supporting Information

Magnetic Fluorescence Molecularly Imprinted Polymer Based on FeO_x/ZnS Nanocomposites for Highly Selective Sensing of Bisphenol A

1. Synthesis of Amino-Modified ZnS: Mn^{2+} QDs

To a 100 mL three-necked flask, 3.6 g $ZnSO_4{\cdot}7H_2O$, 0.2 g $MnCl_2{\cdot}4H_2O$ and 50 mL deionized water were added. The mixture was stirred under the protection dry nitrogen gas at room temperature for 30 minutes, and then 10 mL aqueous solution of $Na_2S{\cdot}4H_2O$ (3.0 g) was slowly dropwise added. The above solutions were kept stirring for 3 hours at 120 °C, and then 0.3 g mercaptoethylamine was added. The mixture was then stirred for another 10 hours under dark condition. The resultant ZnS: Mn^{2+} QDs@MEA was precipitated with methanol, separated by centrifuging, and dried under vacuum for further use.

2. Synthesis of Carboxyl-Functionalized FeO_x MNPs

In brief, 1.95g $FeCl_3{\cdot}6H_2O$, 0.6g $Na_3Cit_2{\cdot}H_2O$ were dissolved in 60 mL ethylene glycol and stirred for 30 minutes. Then, 3.6 g NaAc was added and persistent stirring until a homogeneous rufous solution was obtained. The solution was transferred into the Teflon-lined stainless-steel autoclave and heated at 200 °C for 12 h. The resultant black products were separated by magnet, followed by washing with water and ethanol to remove unreacted reagents, and dried under vacuum for use.

3. The Quantum Yields (QYs) of the FeO_x/ZnS NPs were Calculated According to the Following Equation S1:

$$\phi_x = \phi_s \lfloor A_s / A_x \rfloor [\ln t_x / \ln t_s][\eta_x / \eta_s]^2 \quad \text{(S1)}$$

Where, Φ is the quantum yield, *A* is absorbance at the excitation wavelength, Int is the area under the emission peak, and η is the refractive index of the solvent. The subscripts s and x denote the standard and QDs, respectively. Rhodamine B (QY = 69% in ethanol) was used as a standard for FeOx/ZnS NPs. The quantum yield (QY) of the FeO_x/ZnS NPs was 20.6% in ethanol.

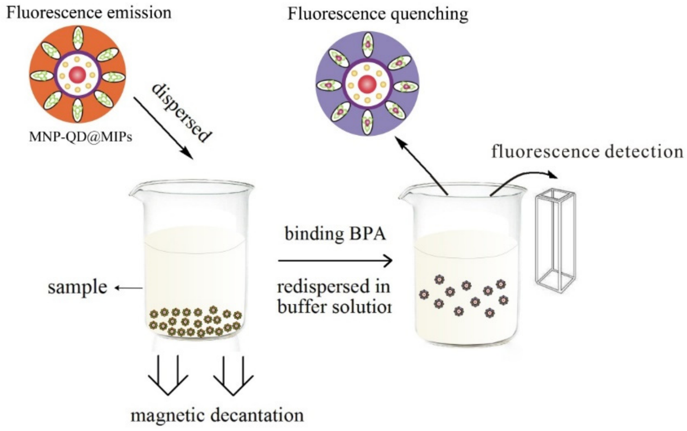

Figure S1. The strategy by using molecularly imprinted FeO_x magnetic nanoparticles and ZnS: Mn^{2+} QDs nanocomposites (MNP-QD@MIPs) for detection of bisphenol A.

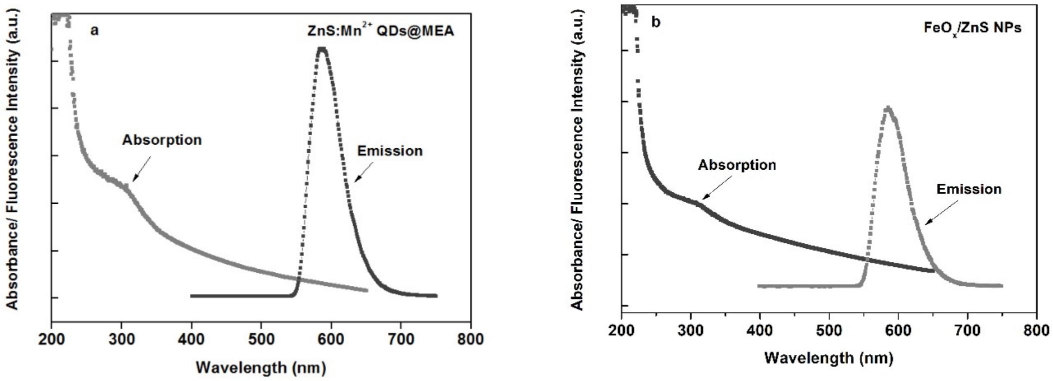

Figure S2. Absorption spectrum and fluorescence spectrum of (**a**) ZnS: Mn^{2+} QDs@MEA and (**b**) FeO_x/ZnS NPs, respectively.

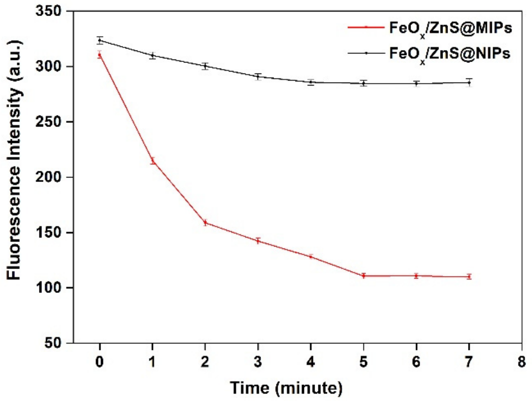

Figure S3. Respond time of FeO_x/ZnS@MIPs and FeO_x/ZnS @NIPs.

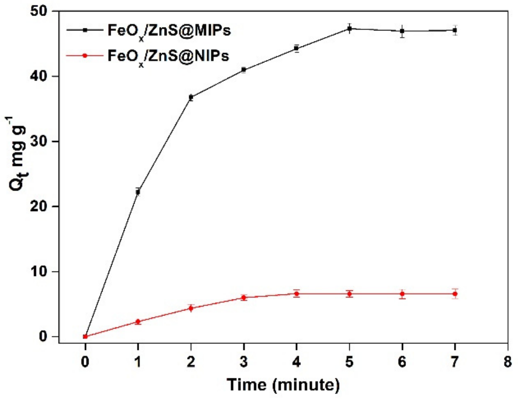

Figure S4. Binding kinetics of FeO_x/ZnS @MIPs and FeO_x/ZnS @NIPs.

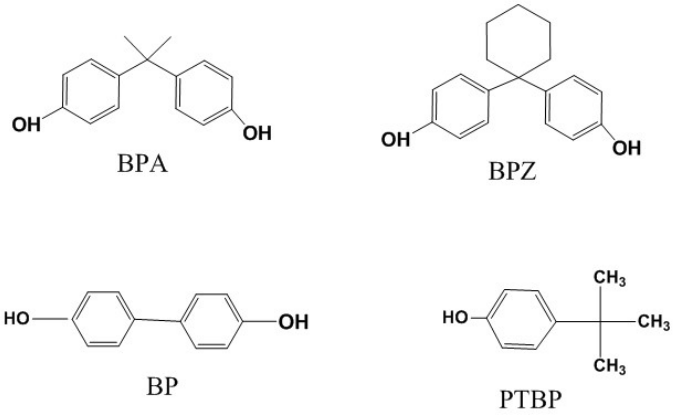

Figure S5. The structural of BPA and its analogs.

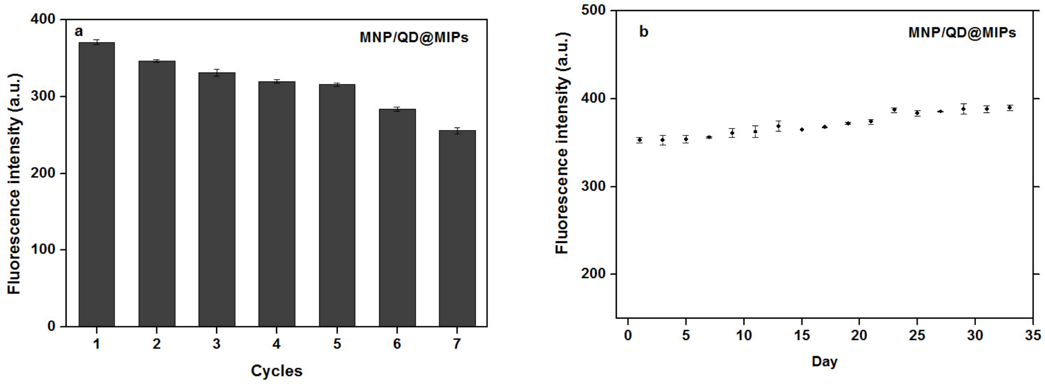

Figure S6. (**a**) Reusability of fluorescence stability of FeO_x/ZnS@MIPs and (**b**) Fluorescence stability of FeO_x/ZnS@MIPs.

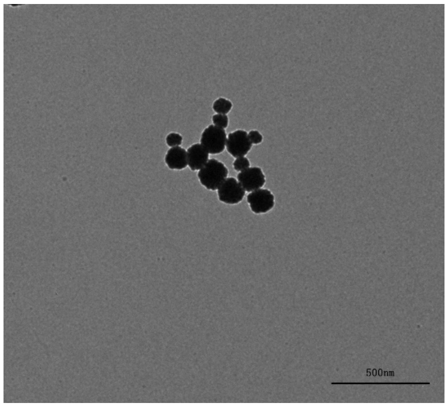

Figure S7. TEM image of carboxyl-functionalized MNPs nanoparticles.

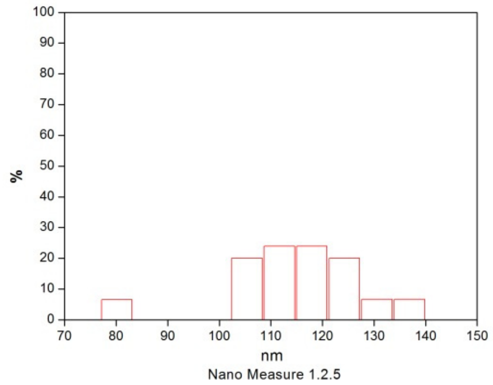

Figure S8. Particle size distribution of carboxy functionalized MNPs.

The TEM image and particle size distribution of the carboxyl-functionalized MNPs nanoparticles are shown in Figure S7 and S8, respectively. Figure S7 showed that the carboxyl-functionalized MNPs nanoparticles have spherical structure, slightly aggregation, and the mean diameter was 117.5 ± 12.4 nm.

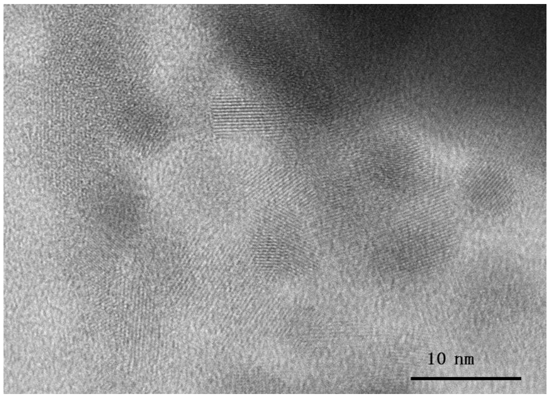

Figure S9. TEM image of ZnS: Mn^{2+}@MEA QDs.

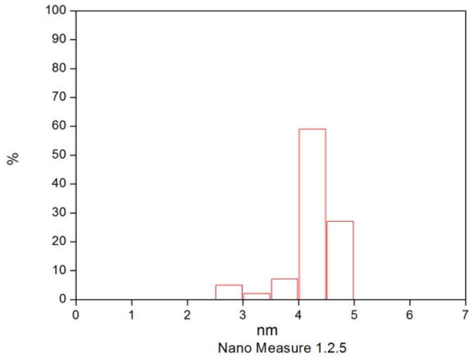

Figure S10. Particle size distribution of ZnS: Mn^{2+}@MEA QDs.

The TEM image and particle size distribution of the ZnS: Mn^{2+}@MEA QDs are shown in Figure S9 and S10, respectively. As shown in Figure S9, the obtained ZnS: Mn^{2+} QDs@MEA show a well crystallized, uniform and spherical morphology, and with a mean diameter of approximately 4.6 nm.

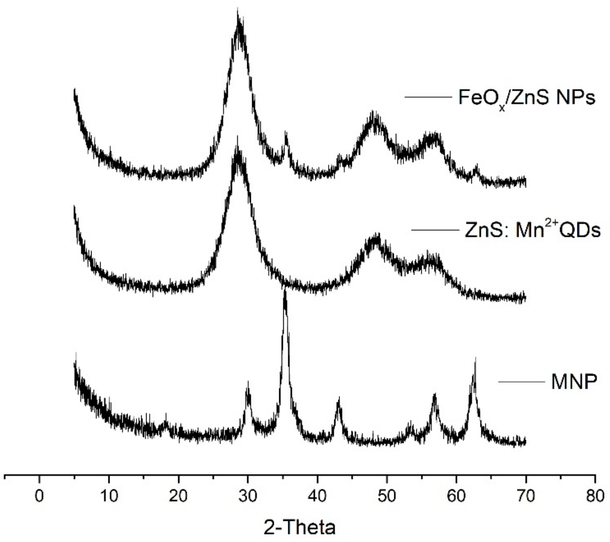

Figure S11. XRD of FeO_x/ZnS NPs, ZnS QDs and MNPs.

The structure and composition of the synthesized FeO_x nanoparticles and ZnS NPs and FeO_x/ZnS NPs were further analysis by X-ray diffractometer. As shown in Figure S11 the XRD pattern of FeO_x/ZnS NPs showed typically broaddiffraction peaks at 30.1°, 35.4°，56.9°，62.5°, which corresponded to（220, (200), (511) and (440) planes of the cubic Fe_3O_4 phase (JCPDS No. 19-0629). At the same time, the peaks at 28.6°,47.6°, 56.3° of MNP/QD NCs corresponded to (111), (220), (311) planes of the cubic (zinc blende) ZnS (JCPDS No. 65-1691). The results of XRD suggested that the ZnS: Mn^{2+} QDs@MEA and FeO_x@COOH were successful conjugated through an EDC/NHS reaction process. XRD analysis results could confirm the successful wrap of ZnS round the MNPs.

Table S1. The quenching constant, imprinting factors, and selectivity coefficients of BPA, BPZ, BP and PTBP for MNP/QD@MIPs and MNP/QD@NIPs.

	$K_{sv,MIP}$	$K_{sv,NIP}$	IF	SC
BPA	0.02451	0.00219	11.19	
BPZ	0.0036	0.00245	1.47	7.61
BP	0.00381	0.00229	1.66	6.74
PTBP	0.00321	0.00231	1.39	8.06
